# In action—an early warning system for the detection of unexpected or novel pathogens

**DOI:** 10.1093/ve/veab085

**Published:** 2021-09-25

**Authors:** Pauline Dianne Santos, Ute Ziegler, Kevin P Szillat, Claudia A Szentiks, Birte Strobel, Jasmin Skuballa, Sabine Merbach, Pierre Grothmann, Birke Andrea Tews, Martin Beer, Dirk Höper

**Affiliations:** Friedrich-Loeffler-Institut, Federal Research Institute for Animal Health, Institute of Diagnostic Virology, Südufer 10, Greifswald, Insel Riems 17493, Germany; Friedrich-Loeffler-Institut, Federal Research Institute for Animal Health, Institute of Novel and Emerging Infectious Diseases, Südufer 10, Greifswald, Insel Riems 17493, Germany; German Centre for Infection Research, Partner Site Hamburg-Lübeck-Borstel-Riems, Südufer 10, Greifswald, Insel Riems 17493, Germany; Friedrich-Loeffler-Institut, Federal Research Institute for Animal Health, Institute of Diagnostic Virology, Südufer 10, Greifswald, Insel Riems 17493, Germany; 4Department of Wildlife Diseases, Leibniz-Institute for Zoo- and Wildlife Research (IZW), Alfred-Kowalke-Straße 17, Berlin 10315, Germany; Chemical and Veterinary Investigations Office Karlsruhe (CVUA Karlsruhe), Weissenburgerstrasse 3, Karlsruhe 76187, Germany; Chemical and Veterinary Investigations Office Karlsruhe (CVUA Karlsruhe), Weissenburgerstrasse 3, Karlsruhe 76187, Germany; State Institute for Chemical and Veterinary Analysis (CVUA) Westfalen, Zur Taubeneiche 10-12, Arnsberg 59821, Germany; Practice for Zoo, Game and Wild Animals, Lintiger Str. 74, Geestland 27624, Germany; Friedrich-Loeffler-Institut, Federal Research Institute for Animal Health, Institute of Infectology, Südufer 10, Greifswald, Insel Riems 17493, Germany; Friedrich-Loeffler-Institut, Federal Research Institute for Animal Health, Institute of Diagnostic Virology, Südufer 10, Greifswald, Insel Riems 17493, Germany; Friedrich-Loeffler-Institut, Federal Research Institute for Animal Health, Institute of Diagnostic Virology, Südufer 10, Greifswald, Insel Riems 17493, Germany

**Keywords:** high-throughput sequencing (HTS), early warning system, metagenomics, Germany, *Peribunyaviridae*, *Reoviridae*, outbreak, bird, mosquitos, Umatilla virus, Hedwig virus

## Abstract

Proactive approaches in preventing future epidemics include pathogen discovery prior to their emergence in human and/or animal populations. Playing an important role in pathogen discovery, high-throughput sequencing (HTS) enables the characterization of microbial and viral genetic diversity within a given sample. In particular, metagenomic HTS allows the unbiased taxonomic profiling of sequences; hence, it can identify novel and highly divergent pathogens such as viruses. Newly discovered viral sequences must be further investigated using genomic characterization, molecular and serological screening, and/or *invitro* and *invivo* characterization. Several outbreak and surveillance studies apply unbiased generic HTS to characterize the whole genome sequences of suspected pathogens. In contrast, this study aimed to screen for novel and unexpected pathogens in previously generated HTS datasets and use this information as a starting point for the establishment of an early warning system (EWS). As a proof of concept, the EWS was applied to HTS datasets and archived samples from the 2018–9 West Nile virus (WNV) epidemic in Germany. A metagenomics read classifier detected sequences related to genome sequences of various members of *Riboviria*. We focused the further EWS investigation on viruses belonging to the families *Peribunyaviridae* and *Reoviridae*, under suspicion of causing co-infections in WNV-infected birds. Phylogenetic analyses revealed that the reovirus genome sequences clustered with sequences assigned to the species *Umatilla virus* (UMAV), whereas a new peribunyavirid, tentatively named ‘Hedwig virus’ (HEDV), belonged to a putative novel genus of the family *Peribunyaviridae*. In follow-up studies, newly developed molecular diagnostic assays detected fourteen UMAV-positive wild birds from different German cities and eight HEDV-positive captive birds from two zoological gardens. UMAV was successfully cultivated in mosquito C6/36 cells inoculated with a blackbird liver. In conclusion, this study demonstrates the power of the applied EWS for the discovery and characterization of unexpected viruses in repurposed sequence datasets, followed by virus screening and cultivation using archived sample material. The EWS enhances the strategies for pathogen recognition before causing sporadic cases and massive outbreaks and proves to be a reliable tool for modern outbreak preparedness.

## 1. Introduction

Based on our response to the 2009 H1N1 pandemic, the World Health Organization and other authorities warned that ‘the world is ill-prepared to respond to a severe influenza pandemic or to any similarly global, sustained and threatening public-health emergency’ (World Health Organization Director-General 2011; Fineberg 2014). This conclusion still stands for the 2013–6 Western African Ebola virus disease epidemic (Ross, Crowe, and Tyndall 2015) and the ongoing coronavirus disease 2019 pandemic, causing more than 4 million deaths to date (World Health Organization 2021). Emerging infectious disease preparedness involves activities that enhance the prevention and control of (re)-emerging pathogens to protect public and animal health (Brookes et al., 2015). Scientific and public health communities often focus on reactive approaches in handling emerging global epidemics (Bloom, Black, and Rappuoli 2017; Greenberger 2018; Kelly et al., 2020), such as Disease X. However, the over-reliance on reactive responses can have a devastating impact on human lives and the global economy.

Investigating viral diversity in wildlife reservoirs is a building block for preparedness for future epidemics. The discovery of novel viruses in animal reservoirs can improve the rapid identification of emerging pathogens and their ecological niche, allowing risk reduction strategies for spillover events and diminishing the severity of emerging outbreaks (Epstein and Anthony 2017). However, as the vast majority of the wildlife virome is still unknown, hunting novel viruses remains an interminable task (Carroll et al., 2018; Carlson 2020). Traditionally, cell culture techniques were applied for virus discovery (Hsiung 1984; Leland and Ginocchio 2007). However, the vast number of viruses are nonculturable; thus, exploration of viral diversity necessitates culture-independent techniques, such as genomic sequencing (Gao and Moore 1996; Mokili, Rohwer, and Dutilh 2012; Mettenleiter 2017). Carroll et al. (2018) estimated that several billion dollars would be needed to unravel all unknown viral species in mammalian and avian hosts by using genomic sequencing.

Genomic sequencing techniques—such as the combined consensus polymerase chain reaction (cPCR) and deep sequencing, and metagenomic high-throughput sequencing (mHTS)—enable high-throughput discovery and taxonomic identification of novel viruses in a sample. The combined cPCR and deep sequencing approach utilizes degenerate primers to amplify conserved regions shared among the members of a viral group flanking their variable regions. This approach is cheaper and more sensitive than mHTS, but it can fail to recognize highly divergent sequences of novel viruses (Chiu 2013). However, mHTS enables hypothesis-free sequencing of all nucleic acids in a given sample, including genomes from completely unknown and highly divergent pathogens (Gu, Miller, and Chiu 2019). mHTS is widely used as a tool for virus discovery in humans (Wylie et al. 2012), wildlife reservoirs (Epstein et al., 2010; Quan et al., 2013b; Sachsenröder et al., 2014; Vibin et al., 2020), domestic animals (Blomström et al., 2009; Bennett et al., 2020; Cibulski et al., 2020), blood-sucking vectors (Brinkmann, Nitsche, and Kohl 2016), and other arthropods (Cox-Foster et al., 2007; Käfer et al., 2019), as well in determining etiological agents in clinical cases and outbreaks (Briese et al., 2009; Hoffmann et al., 2012; Pfaff et al., 2017; Schlottau et al., 2018; Chiu and Miller 2019; Forth et al., 2019; Chen et al., 2020). Several studies also discovered new viruses via data mining of publicly available transcriptome data (Schomacker, Collins, and Schmidt 2004; Basler, García-Sastre, and Palese 2005). However, Canuti and van der Hoek (2014) emphasized the importance of virus characterization after sequence-based discovery to understand their relevance in public and veterinary health. These follow-up investigations include epidemiological analyses using molecular and serological diagnostic tools alongside *invitro* and *invivo* characterization of newly discovered viruses.

Here, we introduce an early warning system (EWS) for the detection of novel or unexpected pathogens and applied it in a pilot study. This EWS takes advantage of HTS datasets from previous studies generated from libraries constructed using only untargeted shotgun sequencing procedures, i.e. datasets derived from generic sequencing approaches. These datasets are analyzed using a metagenomics read classifier to detect sequences that point toward the presence of potential pathogens in the samples from which these reanalyzed datasets are derived. After the initial detection of a potential pathogen, diverse analyses can be initiated, from in-depth genomic characterization of the detected potential pathogen through the design of reverse transcription quantitative PCR (RT-qPCR) assays and subsequent screening of additional samples in the attempt of pathogen isolation. In a pilot study, we successfully applied this EWS to datasets that were generated for the analysis of West Nile viruses (WNV) from the 2018–9 epidemic in Germany (Ziegler et al., 2019, 2020), in which we detected at least two novel or unexpected viruses.

## 2. Materials and methods

### 2.1 Overview of the EWS workflow

**Figure 1.**
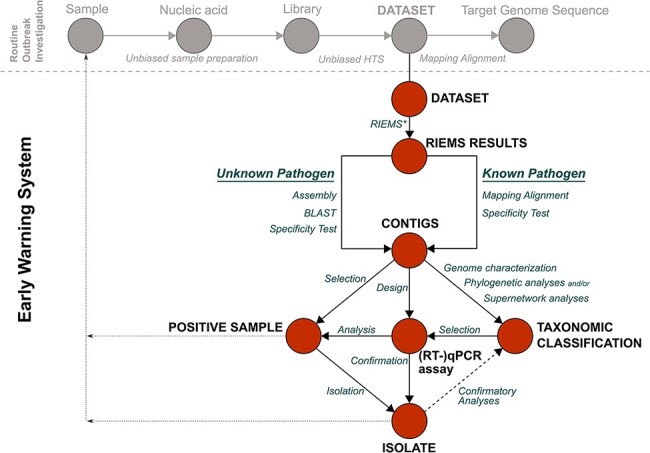
EWS for the detection and characterization of novel and co-infecting pathogens using archived unbiased HTS. Arrowheads indicated the flow of the pipeline. The gray circles and arrows indicate a routine outbreak investigation workflow to acquire the genome sequences of pathogens of interest. The EWS starts at the dataset. Blue text indicates the applied methods, and red dots indicate the results of the methods. For further details, please refer to the text. *RIEMS—the metagenomics read classifier used in this study.

Figure 1 outlines the process of the EWS. At the heart of the EWS is the detection of unexpected or novel pathogens by metagenomics analysis of datasets that were, for instance, generated during a routine outbreak investigation (depicted in gray). The datasets used for this purpose must have been generated with a generic workflow (Wylezich et al., 2018), i.e. a workflow that does not include any steps for targeting the sequencing like PCR (Quick et al., 2016; Oude Munnink et al., 2020) or target enrichment by capture approaches (Depledge et al., 2011; Wylezich et al., 2021). In more detail, the EWS starts with the taxonomic classification of all reads of the datasets using a metagenomics read classifier; here, the Reliable Information Extraction from Metagenomic Sequence datasets (RIEMS) software (Scheuch, Höper, and Beer 2015) was used. Depending on the initial taxonomic binning results (‘known’ but unexpected or ‘unknown’ pathogens identified), different confirmatory data analyses are applied. For known unexpected pathogens, additional analyses start by mapping along available reference sequences. For unknown pathogens, i.e. for which no suitable reference sequences are available, this starts with genome sequence assembly and BLAST (Basic Local Alignment Search Tool; Altschul et al., 1990). Regardless of the initial way, the generated sequences (labeled ‘contigs’ in Fig. 1) are used for targeted investigations toward the detected potential pathogen. Most importantly, in every case the actual presence of the detected potential pathogen needs to be confirmed. Hence, these targeted follow-up investigations can include, but are not limited to, (i) the selection of published or the design of new specific RT-qPCR assays for the confirmation of the presence of the pathogen and screening in samples from ongoing surveillance and in archived samples; (ii) qPCR-based selection of additional samples for the generation of additional (whole-genome) sequence information of the detected pathogen; (iii) bioinformatics analyses for genomic characterization including phylogenetic analyses; and (iv) pathogen isolation attempts. Isolated pathogens provide further possibilities for follow-up studies and could again be used for completing the genome sequence, functional analyses, or serologic screening and neutralization studies.

### 2.2 Data and *in silico* procedures

#### 2.2.1 Data

For the performed pilot study, datasets generated for outbreak investigations of the 2018–9 WNV epidemic in Germany were utilized (Ziegler et al., 2019, 2020), each comprising between 2E + 05 and 1.2E + 07 reads. This represents the ‘routine outbreak investigation’ in Fig. 1. Information on the used datasets and the samples from which these datasets originated is summarized in Supplementary Table S1.

#### 2.2.2 Data analyses

As outlined above, the available HTS datasets were analyzed using the metagenomics read classifier RIEMS (Scheuch, Höper, and Beer 2015) for the initial taxonomic classification of the sequence reads. For the confirmation of the initial taxonomic classification, either all reads were mapped along a suitable available sequence from the INSDC databases or reads classified to the superkingdom ‘Virus’ were assembled together with reads that remained unclassified. The resulting contigs as well as remaining singleton reads were analyzed using BLAST. In addition, to rule out cross-contaminations, all positive results were cross-checked with the virus content of samples processed in parallel. For all mappings and assemblies, the Newbler software (v 3.0, Roche/454 Life Sciences) was used. Sequence similarity searches were performed using BLAST ((Altschul et al., 1990); https://blast.ncbi.nlm.nih.gov/Blast.cgi last accessed: 21 September 2021) and the respective databases. The open reading frames (ORFs) of contigs were predicted and translated using Geneious Prime^®^ 2019.2.3 (Biomatters, Auckland, New Zealand). Online bioinformatics tools were used to characterize assembled sequences. Conserved protein motifs were identified using MOTIF Search ((Ogiwara et al., 1996); https://www.genome.jp/tools/motif/ last accessed: 21 September 2021) based on the Pfam (Finn et al., 2014), NCBI-CDD (Marchler-Bauer et al., 2013), and PROSITE Pattern (Sigrist et al., 2013) databases. Signal peptide sequences, glycosylation sites, and putative transmembrane domains were predicted using SignalP-5.0 Server ((Almagro Armenteros et al., 2019); http://www.cbs.dtu.dk/services/SignalP/ last accessed: 21 September 2021), NetNGlyC 1.0 server ((Gupta and Brunak 2002); http://www.cbs.dtu.dk/services/NetNGlyc/ last accessed: 21 September 2021), and TMHMM Server v. 2.0 ((Krogh et al., 2001); http://www.cbs.dtu.dk/services/TMHMM/ last accessed: 21 September 2021), respectively.

Primers and probes for RT-qPCR assays (Table 1) were designed with Primer3 version 2.3.7 (Untergasser et al., 2012) implemented in Geneious. Amino acid sequences were aligned using MAFFT v.7.450 (Katoh and Standley 2013) and BLOSUM62 (Henikoff and Henikoff 1992) as the similarity matrix, and these alignments were visually inspected in Geneious. Maximum likelihood phylogenetic trees with 100,000 ultrafast bootstraps (Minh, Nguyen, and von Haeseler 2013) were calculated in IQ-TREE 1.6.8 (Nguyen et al., 2014) with the best-fit model defined using ModelFinder (Kalyaanamoorthy et al., 2017). Trees were visualized in FigTree v1.4.4 (http://tree.bio.ed.ac.uk/software/figtree/ last accessed: 21 September 2021). Consensus and supernetwork trees were calculated using SplitsTree v.4 (Huson and Bryant 2006). The results were visualized with R (v4.0; (R Core Team 2020)) in conjunction with Rstudio (v1.2.5033; (RStudio Team 2019)) and packages ggplot2 (Wickham 2016) and pheatmap (Kolde 2019). Prior to visualizations, datasets were normalized to read per million (RPM) and logarithmically scaled using the following formulae:


$$\begin{equation*}RPM = {{read\ count\ per\ family } \over {total\ number\ of\ sequence\ reads}} \times {10^6}\end{equation*}$$

$$\begin{equation*}lo{g_{10}}RPM = lo{g_{10}}\left( {RPM + 0.7} \right)\end{equation*}$$

**Table 1.** Primers and probes for UMAV- and HEDV-specific real-time quantitative polymerase chain reaction screening. Primers and probes targeting HEDV L segment were designed based on HEDV partial genome sequences (old), while new primers and probes were designed using the HEDV complete coding sequences.

Primer name	Primer sequences (5ʹ– 3ʹ)	*T*_m_ (°C)	Target
118-F_L Hedwig	ATGAAGGCTTGACTGCTGCT	58	HEDV L
294-R_L Hedwig	ACCACTTGTGCTCACTTCGT	58	Segment (old)
161-P_L-6-Fam Hedwig	6-Fam-TGTGCCTCAGACACGATGCTTTTGGC-BHQ-1	69	
136-F_S Hedwig	TGGCTCGGGGAAATCAACTG	60	HEDV S
235-R_S Hedwig	TGTAGGGATGAAAGCGGACTG	61	Segment (new)
177-P_S-Hex Hedwig	HEX-TGCTTTTGGCGTGGTTGTGTGCGA-BHQ-1	73	
124-F_L Hedwig	GATGAAGGCTTGACTGCTGC	59	HEDV L
292-R_L Hedwig	GGATACCACTTGTGCTCACTTC	62	Segment (new)
180-P_L-6-Fam Hedwig	6-FAM-TGCTTTTGGCGTGGTTGTGTGCGA- BHQ-1	67	
Umatilla_Seg1_2196F	TCCATGACTCTTGAGCCTGT	58	UMAV
Umatilla_Seg1_2260P	HEX-TGTCCGGATTCGTTGGCCCTCCA-BHQ-2	68	Segment 1
Umatilla_Seg1_2345R	TGTTTCAATCCTTGCACCGC	58	
Umatilla_Seg5_769F	CGCAACATCGACCAACACAG	60	UMAV
Umatilla_Seg5_814P	6-FAM-TGCTGTCTGCTGGTGAGAGAACACGT-BHQ-1	69	Segment 5
Umatilla_Seg5_862R	TCCATCTCCAAAGTTCGTAGCA	60	

Abbreviations: *T*_m_—melting temperature; F—forward; R—reverse; L—L segment; S—S segment; Seg—segment.

### 2.3 Laboratory procedures

#### 2.3.1 Samples, cell cultures and virus isolation

RNA samples used for the small-scale screening and virus isolation attempts are summarized in Table 2. These samples were from the WNV study by Ziegler et al. (2019, 2020) (Panel 1) and WNV and USUV surveillance from 2018–20 (Panel 2). For virus isolation attempts, virus-positive bird samples were selected based on quantification cycle (*C*_q_) values. Approx. 30 mg of tissue material were homogenized for 2 min at 30 Hz with 5 mm steel beads in 1 ml maintenance medium using a TissueLyser II instrument (QIAGEN, Hilden, Germany). All handling of tissue samples and virus isolation attempts in cell cultures were done under the respective necessary biosafety level.

**Table 2.** Summary of samples utilized for virus screening and virus isolation attempts. Panel 1 includes samples processed using the generic HTS approach in Ziegler et al. (2019, 2020) and panel 2 includes additional archived RNA samples collected in different regions of Germany from 2018 to 2020, which include samples that tested positive and negative for WNV and USUV.

Host	Year	Region	Panel	Number of samples
Bird	2018	Bavaria	1	2
		Berlin	1	2
		Berlin	2	9
		Saxony	1	1
		Saxony-Anhalt	1	2
	2019	Baden-Württemberg	2	3
		Berlin	1	7
		Berlin	2	40
		Brandenburg	1	3
		Mecklenburg-Western Pomerania	2	1
		North Rhine-Westphalia	2	5
		Saxony	1	9
		Saxony-Anhalt	1	8
	2020	Baden-Württemberg	2	3
		Lower Saxony	2	13
		Mecklenburg-Western Pomerania	2	1
		North Rhine-Westphalia	2	6
		Rhineland-Palatinate	2	10
Mammal	2018	Brandenburg	1	1
	2019	Berlin	2	13
		Saxony	1	1

All cell lines used in this study were obtained from the Collection of Cell Lines in Veterinary Medicine (CCLV) at the FLI Isle of Riems. Baby hamster kidney cells (BHK-21, RIE0164) and *Cercopithecus aethiops* kidney cells (Vero B4, CCLV1146; Vero E6 cells, CCLV0929) were cultured in minimal essential medium, supplemented with 10 per cent fetal calf serum (FCS), at 37°C and 5 per cent CO_2_. Mosquito cells from *Aedes albopictus* (C6/36, RIE1299) and midge cells from *Culicoides sonorensis* (KC cells, CCLV1062) were cultured in Eagle’s minimal essential medium, supplemented with 10 per cent FCS at 28°C and 2.5 per cent CO_2_. Cells were seeded 1 day prior to infection. On the day of infection, the cells were washed once with a maintenance medium (supplemented with penicillin, streptomycin, and gentamicin) before they were infected with 100 µl of sample homogenate. After inoculation, the cells were cultured for 3 days (BHK-21) at 37°C, 5 per cent CO_2_, for 4–7 days (Vero E6, Vero B4) at 37°C and 5 per cent CO_2_, or for 7 days (C6/36 or KC cells) at 28°C, 2.5 per cent CO_2_, before they were frozen at −20°C. Crude cell culture extracts from BHK-21 and C6/36 cells were thawed and passaged three times to the same cell line. Further details of cell-culture conditions are summarized in Supplementary Table S9. Where appropriate, host switching between BHK-21 and KC cells and vice-versa was also performed to mimic the natural transmission of arboviruses. All cell cultures were investigated for virus replication by RT-qPCR and cytopathic effects (CPE) in all setups.

#### 2.3.2 Nucleic acid extraction and RT-qPCR

For the preparation of RNA for RT-qPCR, RNA extraction from cell cultures was performed using either Agencourt^®^ RNAdvance™ Tissue kit (Beckman Coulter, Indianapolis, USA) or Qiagen RNeasy^®^ Mini kit (Qiagen, Hilden, Germany) according to manufacturer’s instructions. RT-qPCR assays were performed using the SensiFAST™ Probe^®^ No-ROX One-Step Kit (Bioline Meridian Bioscience, USA) in 20 µl reaction volume. The reaction mixes consisted of 2× SensiFAST™ Probe^®^ No-ROX One-Step Mix, 0.2 µl reverse transcriptase, RNase free water, 0.4 µM each of forward and reverse primers, 0.1 µM probe (Table 1), and 2.5 µl total RNA. Amplification was performed in a CFX96™ Touch Real-Time PCR Detection System (Bio-Rad, Feldkirchen, Germany) using the following program: 10 min at 45°C for reverse transcription, 5 min at 95°C for polymerase activation; 45 cycles of 5 s at 95°C, 20 s at 60°C (with fluorescence detection during this step).

#### 2.3.3 Sequencing

For additional sequencing, libraries were prepared from samples processed from sample disintegration until library preparation as described in Wylezich et al. (2018). Table 3 summarizes the samples and conditions that were used for sequencing. For library preparation, the appropriate platform-specific barcoded adapters were used as indicated in Table 3. Sequencing was done either using an Illumina MiSeq in 300 bp PE mode with MiSeq v3 600 cycle reagent kits (all Illumina Inc., San Diego, CA, USA) or an Ion Torrent S5 XL instrument with Ion 550 chips and chemistry in 200 bp runs (Thermo Fisher Scientific, Waltham, MA, USA).

**Table 3.** Additional samples for generic high-throughput sequencing. This includes the sample processing workflow, the library number, and the sequencing platform.

Sample type	Sample description	Sample processing	Library number	Sequencing platform
Tissue	ED-I-79/18 snowy owl 1 spleen tissue	Wylezich et al., 2018 RNAdvance	lib03211	Illumina
Cell culture supernatant	Second passage of ED-I-93/19 blackbird liver tissue in mosquito C6/36 cells (7 days post-infection)	Wylezich et al., 2018 LBE Buffer + RNAdvance	lib04217	Ion Torrent

## 3. Results and discussion

In the present proof-of-concept study, the EWS outlined above was used to analyze datasets previously generated for outbreak investigations. The initial rationale was to gain additional information from a few samples that were only weakly positive for WNV, the presumptive cause of death of the host animal. In these additional analyses of the generically generated HTS datasets, we detected sequences pointing toward the presence of new potential pathogens. The detection of reads pointing at viruses, bacteria, protozoa, and other parasites shows that datasets derived from generically prepared libraries are suitable for the detection of all classes of pathogens, as previously shown for the applied laboratory workflow (Wylezich et al., 2018, 2019, 2020; Bennett et al., 2020; Ziegler et al., 2020). Amongst others, sequence reads potentially belonging to bacteria (families *Pasteurellaceae*, *Clostridiaceae*, *Vibrionaceae*, *Shewanellaceae*, *Enterococcaceae*, *Campylobacteraceae*, *Helicobacteraceae*, and *Hafniaceae*), protozoa (families *Plasmodiidae*, *Eimeriidae*, *Babesiidae*, *Sarcocystidae*, and *Trypanosomatidae*), and other parasites (*Taeniidae*, *Ascarididae*, *Strongyloididae*, and *Schistosomatidae*) that probably infected these vertebrate hosts were detected (Supplementary Table S2). The sequence reads of bacterial and parasitic origin can be analyzed in the EWS downstream analysis. However, here we focused on viral sequence reads and attempted in-depth analyses of datasets for virus detection and characterization.

Since potentially new viruses were detected in the initially analyzed datasets, the same EWS strategy was applied to all remaining datasets of the WNV outbreak investigation. Besides several weak hits, we were able to assemble and characterize complete coding sequences of three unexpected viruses: Alphamesonivirus 1, Umatilla virus (UMAV), and an unclassified member of the family *Peribunyaviridae*. We developed molecular diagnostic assays for two putative viral vertebrate pathogens and screened for these viruses in archived samples providing preliminary information on their hosts and potential tissue tropism. Moreover, we were able to isolate one of the viruses *invitro*.

### 3.1 Overview of the initial screening results

**Figure 2.**
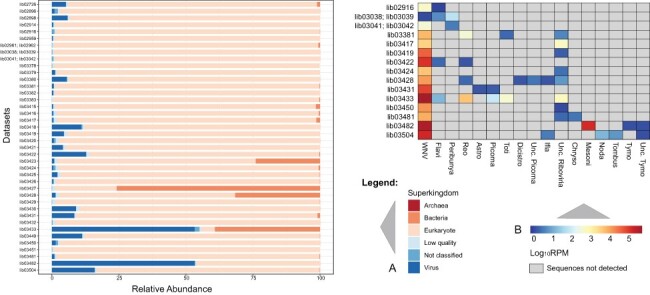
Distributions of sequence reads within generic HTS datasets derived from the 2018 to 2019 WNV epidemic in Germany according to taxonomic classification. (A) Relative abundance of each superkingdom per dataset. (B) Relative abundance of each virus taxon in selected generic HTS datasets. Frequencies of each virus taxon were normalized using the formulae in Section 2.2.2. Light gray boxes indicate that a specific virus sequence was not detected in the dataset. Abbreviations: WNV—West Nile virus; Flavi—*Flaviviridae* (except WNV); Peribunya—*Peribunyaviridae*; Reo—*Reoviridae*; Astro—*Astroviridae*; Picorna—*Picornaviridae*; Toti—*Totiviridae*; Dicistro—*Dicistroviridae*; Unc. Picorna—Unclassified *Picornavirales*; Ifla—Iflaviridae; Unc. Riboviria—Unclassified *Riboviria*; Chryso—*Chrysovirida*e; Mesoni—*Mesoniviridae*; Noda—*Nodaviridae*; Tombus—*Tombusviridae*; Tymo—*Tymoviridae*; Unc. Tymo—Unclassified *Tymovirales.*

Overall, following the EWS strategy, we detected non-WNV viral sequence reads in 15 out of 40 analyzed HTS datasets. Table 4 and Fig. 2 summarize the findings of these initial metagenomics analyses. As shown in Fig. 2A, expectedly (since tissue samples were analyzed and neither host depletion nor any enrichment was performed during sample preparation) the vast majority of the reads were classified as being of eukaryotic origin. Despite the low abundance of viral and unclassified sequence reads in most datasets (Fig. 2A), paired with a dominance of WNV among viral reads (Fig. 2B), a number of reads potentially belonging to other viruses than WNV were identified. While in datasets from cell cultures inoculated with *Culex pipiens* pools, only reads representing viruses that are commonly reported in invertebrate hosts (families *Chrysoviridae, Mesoniviridae, Nodaviridae, Tombusviridae, Tymoviridae*, and order *Tymovirales*) were detected, we found reads putatively representing the viral families *Peribunyaviridae*, *Reoviridae, Astroviridae, Totiviridae, Dicistroviridae*, and *Flaviviridae* (other than WNV) in datasets derived from bird samples. In addition, in both bird and mosquito datasets reads pointing toward the presence of viruses belonging to the family *Iflaviridae* or other members of *Riboviria* were present. Noteworthily, the results from samples inoculated in cell cultures, such as those obtained from the *C.pipiens* pools (datasets lib03481, lib03482, and lib03504), should be interpreted carefully due to the possibility of false-positive and false-negative results. These might result from, e.g. enrichment of adventitious or commensal viruses or inability to cultivate nonculturable viruses in a sample. Employing a broader diversity of cell lines and minimizing the storage period of samples prior to isolation might help increase the success rate of virus isolation.

**Table 4.** The unexpected viral sequence reads detected in several generic HTS datasets sequenced from the 2018 to 2019 WNV epidemic in Germany and their closest relatives. Detailed information regarding the number of sequence reads and length of assembled contigs per closest blastx hits are described in Supplementary Table S3.

Dataset	Host	Taxonomic classification	Closest relative	Number of reads
lib02916	Tawny Owl	*Flaviviridae*	Rodent pestivirus	1
lib03038;	Snowy Owl #1	*Flaviviridae*	USUV	50
lib03039		*Peribunyaviridae*	ASUMV; low aa sequence identities with Thimiri virus; Guama virus	728
lib03041; lib03042	Snowy Owl #2	*Peribunyaviridae*	ASUMV; low aa sequence identities with Belmont virus; Mapputta virus	32
lib03381	Blue Tit #1	*Reoviridae*	UMAV (7 segments, 1 segment with low aa sequence identities); KHV (2 segments); stretch lagoon orbivirus (1 segment)	1654
		*Totiviridae*	Trichoderma koningiopsis totivirus 1	6
		*Riboviria*	Hubei toti-like virus 6; Wuhan insect virus 27	17
lib03417	Goshawk #3	*Riboviria*	Hubei toti-like virus 6	560
lib03419	Goshawk #5	*Riboviria*	Hubei toti-like virus 6	1
lib03422	Great Tit #1	*Flaviviridae*	USUV	2
		*Reoviridae*	Stretch lagoon orbivirus	1
lib03424	Goshawk #7	*Riboviria*	Hubei toti-like virus 6; Lake Sinai virus 5	2
lib03428	House Sparrow	*Dicistroviridae*	Barns Ness breadcrumb sponge dicistro-like virus 2	1
		*Iflaviridae*	King virus	1
		*Picornavirales*	Antarctic picorna-like virus 1	3
		Reoviridae	Avian orthoreovirus	2
		*Riboviria*	Jingmen tombus-like virus 1, Nadgee virus, Pink bollworm virus 4, Sanxia picorna-like virus 11, Sanxia picorna-like virus 9	8
lib03431	Great Grey Owl #5	*Picornaviridae*	Norway rat kobuvirus 2	3
		*Astroviridae*	Murine astrovirus	2
lib03433	Great Tit #2	*Flaviviridae*	Duck hepacivirus; Theiler’s disease-associated virus; Jogalong virus	3
		*Picornaviridae*	Washington bat picornavirus	17
		*Reoviridae*	UMAV (7 segments, 1 segment with low aa identities); KHV (2 segments); stretch lagoon orbivirus (1 segment)	1062
		*Totiviridae*	*Eimeria* *stiedae* RNA virus 1; *Eimeria tenella* RNA virus 1; *E.brunetti* RNA virus 1; *Linepithema humile* toti-like virus 1; *Trichomonas vaginalis* virus 2	86
		*Riboviria*	*Hubei partiti*-like virus 48, Baker virus, Volivirus, *Hubei orthoptera* virus 4, Cordoba virus, *Hubei picorna*-like virus 71	132
lib03450	Goshawk #8	*Riboviria*	Wilkie narna-like virus 1	1
lib03481	Mosquito Pool #1	*Chrysoviridae*	Eskilstorp virus; Shuangao chryso-like virus 1	4
		*Riboviria*	Hubei chryso-like virus 1	3
lib03482	Mosquito Pool #2	*Mesoniviridae*	Alphamesonivirus 1	242,607
		*Tymoviridae*	Bombyx mori latent virus	1
		*Tymovirales*	Guadeloupe Culex tymo-like virus	1
lib03504	Mosquito Pool #3	*Nodaviridae*	Culex mosquito virus 1	29
		*Picornavirales*	Culex picorna-like virus 1	13
		*Tombusviridae*	Culex-associated Tombus-like virus	12
		*Iflaviridae*	Culex-Iflavi-like virus 4	5
		*Riboviria*	Hubei chryso-like virus 1	1

Most of the previously mentioned viral taxonomic groups were only represented by few sequence reads with low sequence identities when compared to sequences from the databases (Table 4, Supplementary Table S3). Especially unclassified members of *Riboviria* were frequently found in bird datasets (Table 4, Supplementary Table S3). These viruses were previously detected in virome analyses of various invertebrate sample pools collected in China (Shi et al., 2016), and the birds probably obtained these viral sequences from their insect or arthropod diet without being infected by these viruses. In dataset lib03433, a contig was classified to the family *Totiviridae*, having the highest sequence identities with sequences of different species of viruses from apicomplexan hosts (Table 4 and Supplementary Table S3). However, corresponding sequences related to protozoan parasites were not found in dataset lib03433, although, for instance, the protozoan *Eimeria brunetti* is known to cause coccidiosis in birds (Kawahara et al., 2014). In this group of viruses, represented by only a few reads, we also discovered viruses that potentially infect vertebrate hosts (Table 4 and Supplementary Table S3). This group comprises six viruses, namely an avian orthoreovirus (lib03428), an unclassified kobuvirus and an astrovirus (lib03431), an unclassified hepacivirus and a pegivirus (lib03433), and an unclassified pestivirus (lib02916). Although contigs could be assembled in some instances, the information was insufficient for subsequent EWS steps.

Amongst the viruses represented by a low number of reads, we also detected Usutu virus (USUV) in datasets lib03038/lib03039 and lib03422 (Table 4). These findings confirmed the previously reported WNV/USUV co-infections in the animals from which these datasets were derived (Santos et al., 2021). However, we could not detect USUV reads in dataset lib03041/lib03042, which was also derived from a bird that tested positive for both WNV and USUV. In our previous study, viral sequence enrichment and virus-specific multiplex PCR had to be employed to acquire the full genomes of both flaviviruses (Santos et al., 2021). Owing to the previously performed complete analysis, here we did not pursue USUV for EWS downstream analysis. Nevertheless, the low abundance of USUV in these samples caused two true-positive and one false-negative results regarding the presence of USUV. This highlights one potential drawback of this EWS, namely the eventually limited sensitivity. This can on the one hand be caused by the size of the available dataset, as shown in very much detail by Ebinger, Fischer, and Höper (2021). On the other hand, failure to detect can likewise be due to sequencing of less suitable sample matrices for the respective virus, depending on the virus’s tissue tropism.

It is also noteworthy that three different viruses with high abundances were found in different samples. These were subsequently taken to the next level of analysis according to the EWS concept (Fig. 1). First, reads representing the family *Mesoniviridae* with highest identity with Alphamesonivirus 1 sequences were detected in one of the datasets (lib03482) generated from mosquito pools. Second, an unexpected orbivirus that had not been detected in Germany before was found in datasets lib03381 (>1600 reads) and lib03433 (>1000 reads). Third, more than 700 reads pointing toward the presence of an unexpected peribunyavirid were detected in dataset lib03038/lib03039. A few reads representing the same peribunyavirid were also detected in dataset lib03041/lib03042. The subsequent analyses and the obtained results are summarized in the following sections.

### 3.2 EWS follow-up analyses—genomic characterization

#### 3.2.1 Mosquito virus *Alphamesonivirus 1*

The 20,125-nucleotide long contig from dataset lib03482 (mosquito pool #2 inoculated in C6/36 cells) had 99.5 per cent nucleotide identity with an Alphamesonivirus 1 found in *C.pipiens* in Italy (Accession MF281710). Its RNA-dependent RNA polymerase (RdRp) amino acid sequence clustered with other strains of the species Alphamesonivirus 1 (Supplementary Fig. S1). Alphamesonivirus 1 species members are reported in a broad range of mosquito species collected in different parts of the world (Vasilakis et al., 2014) and as a co-infecting agent with Zika virus in the C6/36 cell culture (Sardi et al., 2020). Since this virus has not been associated with disease in vertebrates so far, we stopped the EWS investigation at this point.

#### 3.2.2 Unexpected orbivirus in two wild birds

**Figure 3.**
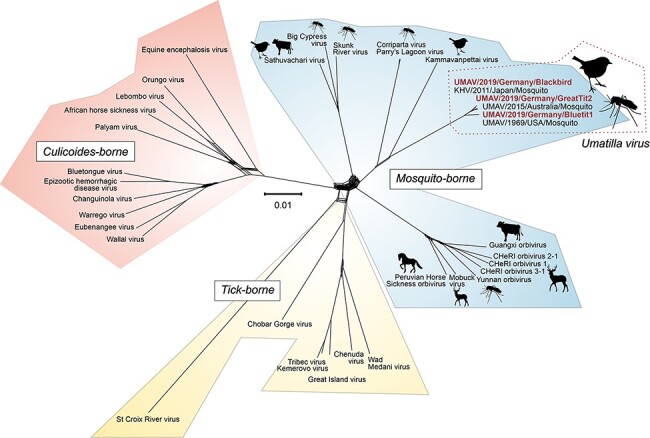
The genus *Orbivirus* supernetwork. This supernetwork analysis is based on ten maximum likelihood trees from representative *Orbivirus* species with complete segments (*n* = 10 segments). Red text indicates UMAV variants detected in this study. Accession numbers of available amino acid sequences from representative members of the genus *Orbivirus* are indicated in Supplementary Table S4. Images were acquired from Pixabay under Pixabay license (https://pixabay.com/service/license/ last accessed: 21 September 2021).

Nearly complete coding sequences of decapartite reovirus genomes were assembled from datasets lib03381 (blue tit) and lib03433 (great tit). In phylogenetic analyses (Fig. 3, Supplementary Fig. S2; Table S4), these genome sequences from Germany clustered with members of the species UMAV, with UMAV strains from the USA forming a separate subcluster. Except for the outer capsid protein (OCP) 1, high amino acid sequence identities among UMAV species were observed for all proteins (Supplementary Table S5). Sequence variations in OCP1 were expected since it is the major virus antigen of the genus *Orbivirus*, inducing specific neutralizing antibodies that distinguish distinct serotypes of each species (Mertens et al., 1989). Interestingly, further variations between the UMAV sequences were detected in their 3′ untranslated regions (3′ UTR). All UMAV except two strains from the USA have deletions in the 3′ UTR of the segments encoding the nonstructural protein 1 and OCP1 (Supplementary Fig. S3). Similar deletions were described before in Koyama Hill virus (KHV) segments in comparison with UMAV strain USA 1969 (Ejiri et al., 2014). These deletions within the 3′ UTR may cause lower levels of viral mRNA expression, as was previously shown for the Bluetongue virus, another member of the genus *Orbivirus* (Boyce, Celma, and Roy 2012). Hence, deletions at the 3′ UTR of NS1 and OCP1 coding segments in these viruses may affect their growth kinetics and pathogenicity.

Phylogenetic analyses (Fig. 3 and Supplementary Fig. S2) and comparison of the amino acid sequences derived from the RdRp and T2 encoding sequences (Supplementary Table S5) imply that according to the demarcation criteria specified for orbiviruses (Attoui et al., 2012), the detected reovirus belongs to the genus *Orbivirus*, species UMAV. In detail, the deduced RdRp sequences of UMAV strains from Germany have ≥37.8 per cent identity with RdRp of other orbiviruses (genus demarcation ≥30 per cent identity), while their deduced T2 sequences exhibit 94 per cent identity with T2 of other members of the UMAV species (species demarcation ≥91 per cent identity).

The species UMAV consists of the four recognized serotypes Umatilla and Llano Seco virus from the USA, Minnal virus from India, and Netivot virus from Israel (Mertens et al., 2005; Belaganahalli et al., 2011). Knowledge regarding the biological characteristics, host range, epidemiology, pathogenicity, and geographical distribution of UMAV species is limited. The aforementioned were mainly isolated from different *Culex* species (Dandawate and Shope 1975; Gubler and Rosen 1976; Karabatsos 1985; Tesh et al., 1986; Tangudu et al., 2019); other members of the species UMAV were detected in and isolated from *Culex* and *Aedes* mosquitoes from Australia (UMAV and stretch lagoon orbivirus, SLOV) and ornithophilic *Culex* mosquitoes in Japan (KHV). The only report of UMAV isolation from vertebrates was in house sparrows (*Passer domesticus*) collected in the USA in 1967 (Karabatsos 1985; Belaganahalli et al., 2011). Serological data suggest that horses, donkeys, and goats are potential vertebrate hosts of SLOV, while neutralizing antibodies against Minnal virus were detected in sera from three human cases in India (Belaganahalli et al., 2011; Centers for Disease Control and Prevention; Cowled et al., 2009; Ejiri et al., 2014; Tangudu et al., 2019).

#### 3.2.3 Novel peribunyavirid in captive snowy owls

To assemble the complete genome for the novel peribunyavirid, tentatively named HEDV as it was detected in datasets derived from captive snowy owls, additional sequence data had to be generated (lib03211). The new dataset was assembled with the preexisting datasets lib03038/lib03039 from the WNV study yielding three segment sequences of lengths 6,965 bases (L segment), 4,606 bases (M segment), and 1,079 bases (S segment).

**Figure 4.**
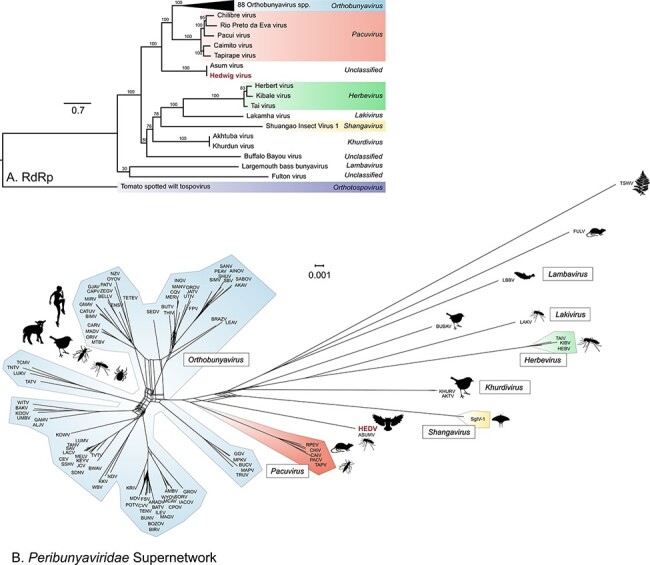
Phylogenetic analyses and supernetwork of representative peribunyavirid, ASUMV and HEDV (printed red). Blue, orange, yellow, green, and uncolored represent the genera *Orthobunyavirus, Pacuvirus, Shangavirus, Herbevirus*, and the recently proposed genera *Lakivirus, Lambavirus*, and *Khurdivirus* or the remaining unclassified members of the family *Peribunyaviridae*. (A) Maximum likelihood tree of the RdRp amino acid sequences. Ultrafast bootstrap analyses with 100,000 replicates supported the tree topology. Representative *Orthobunyavirus* species (*n* = 88) were collapsed into a triangle. Tomato spotted wilt tospovirus was used as an outgroup (violet). (B) Supernetwork of the 3 ML trees calculated for the RdRp, the glycoprotein precursor, and the nucleocapsid protein (for the latter two see Supplementary Fig. 4). Accession numbers of available amino acid sequences from representative members of the family *Peribunyaviridae* and the outlier strain are indicated in Supplementary Table S6. Images were acquired from Pixabay under Pixabay license (https://pixabay.com/service/license/ last accessed: 21 September 2021).

As for the detected reovirus, we started with phylogenetic analysis for classification of the virus. In this analysis, representatives of the four established genera in the family *Peribunyaviridae* were considered, namely *Orthobunyavirus, Herbevirus, Pacuvirus,* and *Shangavirus* (Hughes et al., 2020). In addition, other related unclassified members of the family *Peribunyaviridae* that are listed by the International Committee on Taxonomy of Viruses (Hughes et al., 2020), encompassing Akhtuba virus (Quan et al., 2013a), Fulton virus (Williams et al., 2019), Khurdun virus (Al’kovskhovsk et al., 2013), Lakamha virus (Kopp et al., 2019), and largemouth bass bunyavirus (Waltzek et al., 2019) were included (Supplementary Table S6; results of pairwise sequence comparisons of representative viruses see Supplementary Table S7). Some of these viruses were assigned to the recently proposed new genera *Lakivirus, Lambavirus*, and *Khurdivirus* (Fig. 4 and Supplementary Table S6) (Jens Kuhn, personal communication). Moreover, Asum virus (ASUMV), which was recently reported with only its L segment sequence and not yet designated a member species of the family *Peribunyaviridae* (Pettersson et al., 2019; Hughes et al., 2020), was likewise taken into account, because with 97.2 per cent identity the ASUMV L segment is the closest relative of the HEDV L segment. To include the ASUMV complete genome in phylogenetic analyses, we retrieved the raw sequence dataset harboring its L segment (BioProject PRJNA516782) and mapped ASUMV sequences using HEDV sequences as references. This resulted in three contigs with lengths of 7,161 nucleotides (mean coverage 150), 4,606 nucleotides (mean coverage 298), and 1,235 nucleotides (mean coverage 345), which were included in the phylogenetic reconstruction. As Fig. 4A shows, phylogenetic analysis of the RdRp sequences suggests that HEDV and ASUMV belong to a novel genus of the family since they do not cluster with other established or unclassified peribunyavirid genera (Hughes et al., 2020). In the supernetwork (Fig. 4B), HEDV together with ASUMV branches as a deep rooting lineage within the family *Peribunyaviridae*.

**Figure 5.**
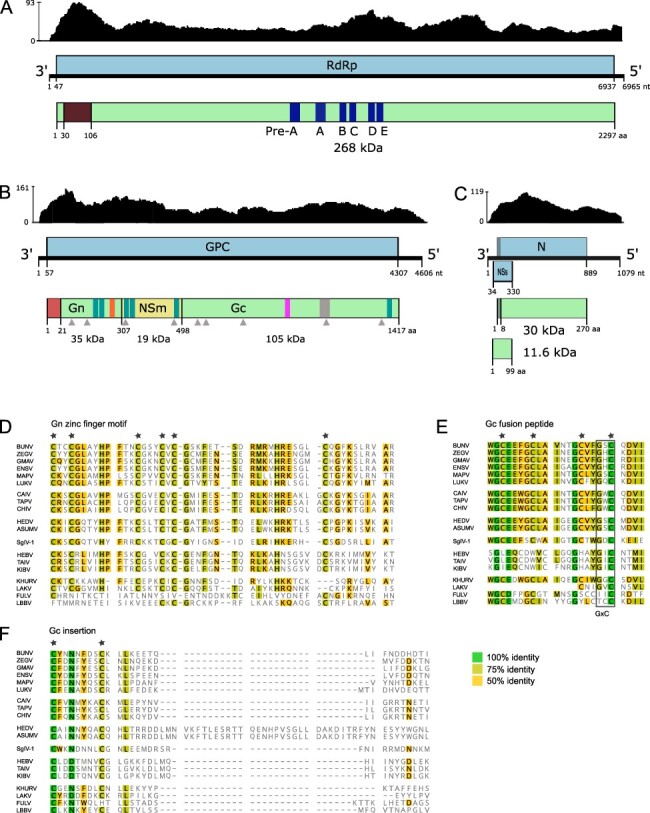
HEDV genome characterization. Schematic organization of HEDV genome segments coding for (A) RdRp, (B) glycoprotein precursor (GPC), and (C) nucleocapsid protein (N). ORFs are shown as light blue boxes, and light green boxes indicate predicted proteins. Genome positions and predicted protein masses are included. The graphs above the genomes show the sequence depths. (A) The brown box indicates the endonuclease domain and dark blue boxes represent pre-motif A and motifs A–E. (B) The red box indicates a putative signal peptide; gray triangles represent predicted N-linked glycosylation sites; teal boxes indicate putative transmembrane domains; the gray box indicates a predicted insertion in the GPC; Gn zinc finger and Gc fusion peptide motif are illustrated with orange and pink boxes, respectively. Alignments of amino acid sequences of conserved regions of (D) the Gn zinc finger motif, (E) the Gc fusion peptide, and (F) the insertion within in GPC. Green, yellow-green, and yellow indicate 100 per cent, 75 per cent, or 50 per cent identity, respectively. Conserved cysteines are indicated by stars above the aligned sequences. Accession numbers of available amino acid sequences from representative members of the family *Peribunyaviridae*are indicated in Supplementary Table S6.

Further in-depth analyses of the tripartite HEDV genome showed an organization very similar to the genera *Orthobunyavirus* and *Pacuvirus*. The HEDV RdRp has the typical motifs within the N-terminal endonuclease domain and conserved sequences for pre-motif A and motifs A–E (Fig. 5A) (Amroun et al., 2017; Kopp et al., 2019). The predicted HEDV nucleocapsid ORF (Fig. 5C) shows two putative in-frame start codons, _80_CUG and _101_AUG. The non-AUG initiation is a natural but rather inefficient start codon. The large proportion of ribosomes will scan past the non-AUG site and initiate at the downstream AUG instead. It was assumed that this leaky scanning mechanism leads to the generation of multiple protein variants with N-terminal extensions or from alternative reading frames (Firth and Brierley 2012). Analysis of the HEDV glycoprotein precursor implies that it is cleaved into Gn, NSm, and Gc proteins (Fig. 5B). However, the HEDV Gn C terminus (VKAI_306_) does not comprise the highly conserved arginine found among the members of the genera *Orthobunyavirus* and *Pacuvirus*. It also differs from the termini of *Herbevirus*, *Shangavirus*, and unclassified viruses of the *Peribunyaviridae* (Fig. 5D). The HEDV glycoprotein precursor comprises a Gn zinc finger motif with conserved cysteine residues found in most peribunyaviridae (Fig. 5D) and a Gc fusion peptide with four conserved cysteine residues found only in *Orthobunyavirus, Pacuvirus*, *Shangavirus*, and Khurdun virus (Fig. 5E). The *Peribunyaviridae* glycoprotein precursor sequence alignment revealed a 26–35 amino acid insertion within the C terminal half of the HEDV Gc protein core region (Fig. 5F), i.e. in the region which mediates cell fusion (Shi et al., 2009).

Altogether, our results show that HEDV is a novel peribunyavirid and a representative species of a presumed novel genus within the family *Peribunyaviridae*. The second member of this putative new genus is its closest relative ASUMV, whose genome was previously only partially assembled from data generated from *C.pipiens* mosquito pools collected in Kristianstad, Sweden, in 2006–7 (Pettersson et al., 2019). Here, we were able to complete the genome of ASUMV, and pairwise alignments of the HEDV and ASUMV genomes demonstrated high nucleotide sequence identities between their L (97.21 per cent), M (96.23 per cent), and S (97.77 per cent) segments. While ASUMV was found in *C.pipiens*, we detected HEDV in two captive snowy owls. Hence, this study adds substantial knowledge regarding the vertebrate host of this potential arbovirus.

### 3.3 RT-qPCR screening—additional positive animals

**Figure 6.**
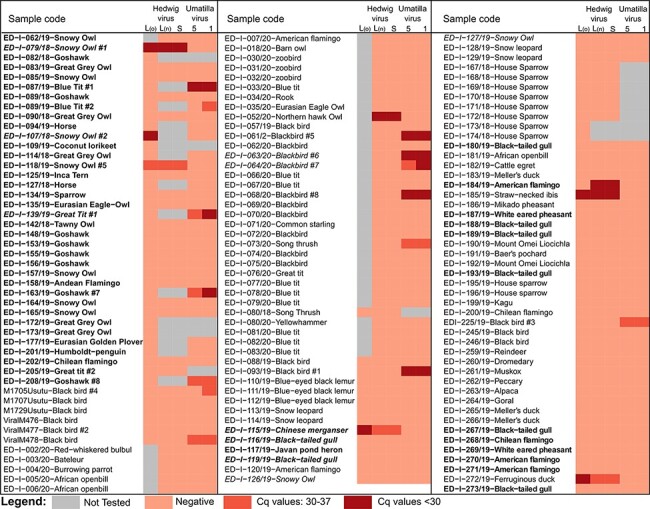
Samples tested using HEDV-specific and UMAV-specific real-time quantitative polymerase chain reaction assays. We designed two primer and probe sets (labeled o = old and n = new) specific to the HEDV L segment. Gray indicates not tested samples. The lightest shade of red indicates negative results, while darker shades of red indicate HEDV- or UMAV-positive samples. Bold indicates WNV-positive samples, italics indicate USUV-positive samples.

Using the assembled UMAV and HEDV sequences, we designed virus specific RT-qPCR assays. With these assays, we screened for UMAV and HEDV in two sample panels collected from 2018 to 2020 composed of RNA extracted from 125 birds and 15 mammals (Table 2 and Supplementary Table S8) with known USUV and WNV status (included in Fig. 6) and some also pretested for other viruses. Unfortunately, RNA from some samples was limited; therefore, we could not test all samples for both HEDV and UMAV. Figure 6 summarizes the results of this small-scale screening. We detected UMAV RNA in fourteen wild birds (*n* = 112), hence, together with the UMAV-positive sample (dataset lib03433), we found UMAV in fifteen birds but not in any mammals (*n* = 13). Eight out of 125 tested bird samples were found positive for HEDV, again, none of the tested mammals (*n* = 15) were positive. Out of the twenty-three UMAV- or HEDV-positive birds, twelve were co-infected with WNV and/or USUV. We found one UMAV-positive and three HEDV-positive birds with confirmed WNV and USUV co-infections (Fig. 6). Where available, we tested different organ samples of the birds (brain, liver, spleen, kidneys, heart, and lungs; Supplementary Table S8). Except for the relatively lower HEDV *C*_q_ value in the snowy owl #1 spleen, no marked tissue tropism was observed for both viruses.

The available necropsy reports of the dead birds were assessed to identify potential symptoms caused by HEDV or UMAV infection. Seven out of the fifteen UMAV-positive wild birds were negative for WNV, USUV, and Hepatitis E virus in RT-qPCR. Necropsy reports of these seven birds described splenomegaly, suggesting an acute infection. Three out of the eight HEDV-positive birds were negative for both WNV and USUV; however, only for two of these a necropsy report was available. According to these reports, the straw-necked ibis had necrotizing dermatitis and weakly pronounced interstitial pneumonia while the ferruginous duck had a swollen spleen and liver, but the suspected cause of death was septicemia due to sand penetration into the subcutaneous tissue of its head.

### 3.4 Virus isolation—UMAV isolated in cell culture

From all available samples, we selected those to attempt virus isolation based on the sequencing results and based on RT-qPCR results for unsequenced samples. While we failed to cultivate HEDV from the selected available organ samples in mammalian and insect cell lines, we successfully isolated UMAV from blackbird #1 liver in C6/36 mosquito cell lines (Supplementary Table S9). Failure to isolate HEDV *invitro* could be caused by the cell lines used, which may not be suitable for HEDV cultivation, or by the long-term storage of organ samples that might have had a negative effect on the viability of HEDV (Leland and Ginocchio 2007; Ørpetveit et al., 2010).

This UMAV strain replicated in C6/36 cells with CPE but did not replicate in BHK-21 cells. Similar observations were reported for KHV, UMAV-IA08, and SLOV-IA08, which replicated and produced CPE in C6/36 cells but not in hamster cell lines (Ejiri et al., 2014; Tangudu et al., 2019). However, other studies reported that two UMAV isolates replicated and produced strong CPE in hamster cell lines (BHK-21 cells and BSR cells, respectively) (Cowled et al., 2009; Belaganahalli et al., 2011). For confirmation of the successful isolation, we generated an Ion Torrent compatible library (lib04217; see Table 3) with RNA isolated from UMAV infected C6/36 cells. We were able to assemble the complete UMAV genome from the generated dataset, which was included in the phylogenetic analyses. Except for the OCP1 encoding segment, this UMAV genome is identical with UMAV genomes from datasets great tit #2 and blue tit #1 (Supplementary Table S5).

## 4. Conclusion

The introduced EWS applies well-established protocols for pathogen discovery and characterization to enable a quasi-hypothesis-free screening for co-infecting and unexpected pathogens in outbreak and surveillance samples without *a priori* knowledge of their presence or even existence. The only hypothesis we employ is that we assume that something might circulate unnoticed and that it can be detected based on its nucleic acids. This only excludes prions. The sensitivity of the EWS for the detection of nucleic acid containing pathogens depends on the pathogen content and dataset size, as shown by the USUV example.

The EWS builds on available datasets generated in the framework of routine outbreak investigations. These datasets must have been generated applying generic and unbiased procedures. Since no extra sample processing is necessary, the required time and resources for protocol development and optimization, but especially for sample collection, preparation, and sequencing can be reduced. This facilitates timely processing, enables integration into routine workflows, and hence helps identify (known) pathogens prior to their emergence.

The three presented examples from the pilot study are a proof of concept for the outlined EWS to detect unexpected or unknown pathogens, showing all possible stages included in the EWS concept. HEDV, detected in snowy owls and other captive birds, together with ASUMV forms a putative novel genus of the family *Peribunyaviridae*. Moreover, we here report the first detection of UMAV within central Europe and its re-detection in birds after more than 50 years. Based on information gained from in-depth genomic characterization, we were able to design RT-qPCR assays and finally isolate UMAV from a blackbird sample. This enables additional follow-up investigations for further virus characterization. The presented screening implies that the detected viruses most likely have circulated unnoticed in Germany. Hence, the EWS can provide necessary information and facilitate the development of diagnostic tools to respond rapidly to emerging infectious diseases before they turn into massive epidemics.

## Supplementary Material

veab085_Supp

## Data Availability

The nucleotide sequences from this study are available from the INSDC databases under study accession PRJEB45282.
