# Supplementary material for: In action—an early warning system for the detection of unexpected or novel pathogens
Source: Virus Evol. 2021 Sep 25;7(2):veab085. doi: 10.1093/ve/veab085 (PMC8542707; doi:10.1093/ve/veab085)
Supplement: veab085_Supp [file veab085_supp.zip › Santos_EarlyWarningSystem_Supplementary_Figures.pdf]

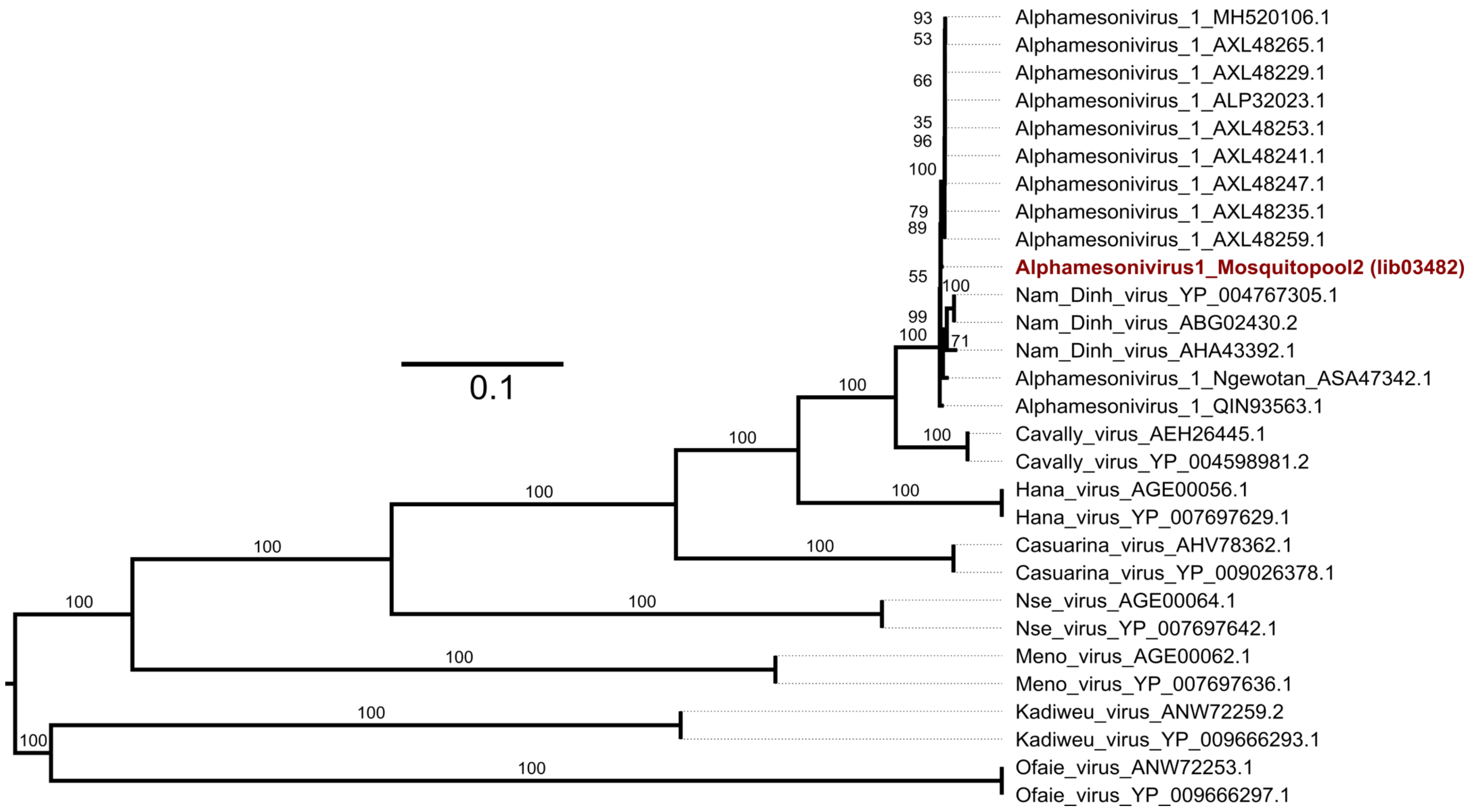

Figure S1. Maximum likelihood phylogenetic tree of the RdRp sequences of members of the family *Mesoniviridae*. Red text indicates the viral genome acquired in this study.

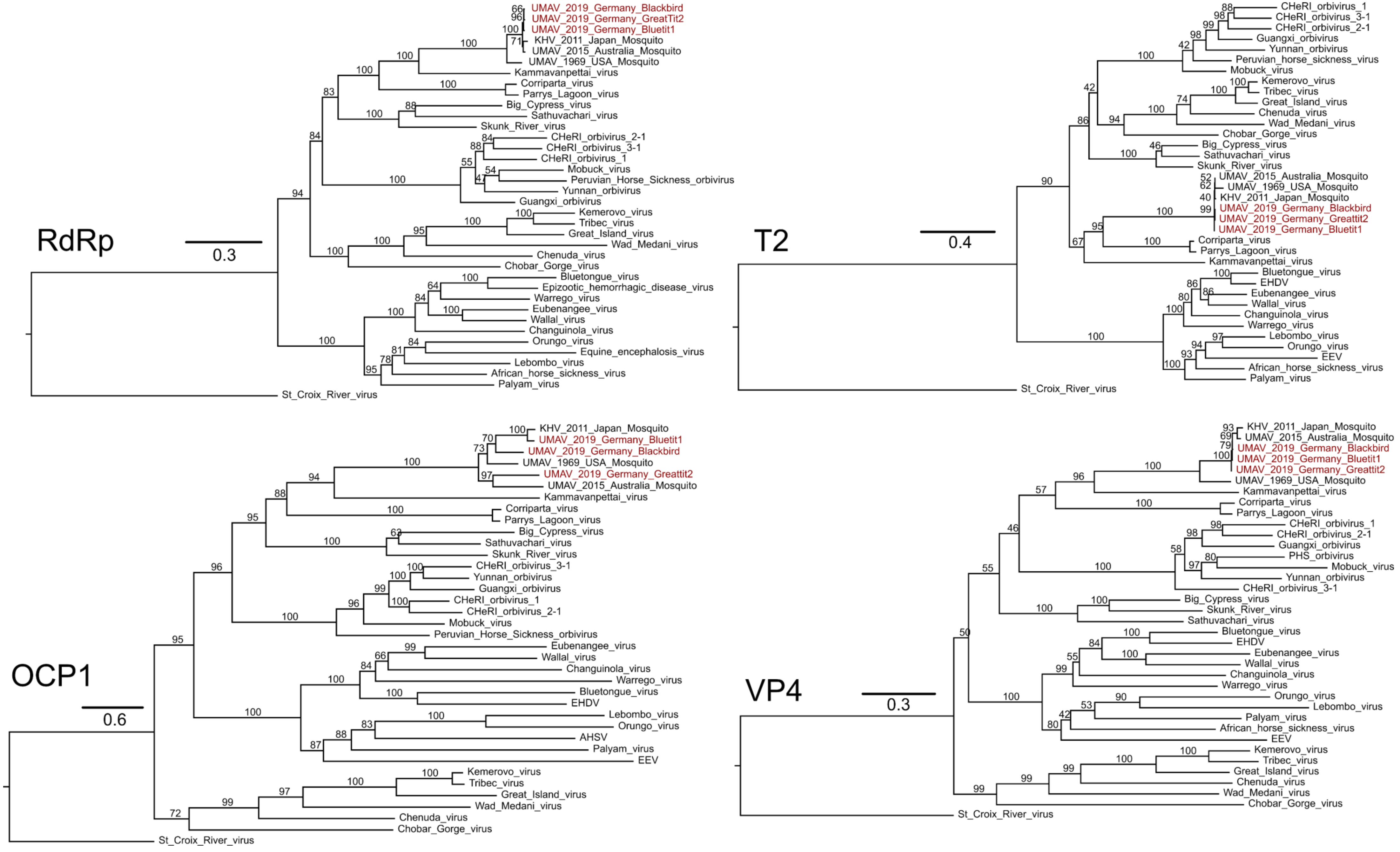
RdRp
0.3
UMAV_2019_Germany_Blackbird
UMAV_2019_Germany_GreatTit2
UMAV_2019_Germany_Bluetit1
KHV_2011_Japan_Mosquito
UMAV_2015_Australia_Mosquito
UMAV_1969_USA_Mosquito
Kammavanpettai_virus
Corriparta_virus
Parrys_Lagoon_virus
Big_Cypress_virus
Sathuvachari_virus
Skunk_River_virus
CHeRI_orbivirus_2-1
CHeRI_orbivirus_3-1
CHeRI_orbivirus_1
Mobuck_virus
Peruvian_Horse_Sickness_orbivirus
Yunnan_orbivirus
Guangxi_orbivirus
Kemerovo_virus
Tribec_virus
Great_Island_virus
Wad_Medani_virus
Chenuda_virus
Chobar_Gorge_virus
Bluetongue_virus
Epizootic_hemorrhagic_disease_virus
Warrego_virus
Eubenangee_virus
Wallal_virus
Changuinola_virus
Orungo_virus
Equine_encephalosis_virus
Lebombo_virus
African_horse_sickness_virus
Palyam_virus
St_Croix_River_virus
T2
0.4
CHeRI_orbivirus_1
CHeRI_orbivirus_3-1
CHeRI_orbivirus_2-1
Guangxi_orbivirus
Yunnan_orbivirus
Peruvian_horse_sickness_virus
Mobuck_virus
Kemerovo_virus
Tribec_virus
Great_Island_virus
Chenuda_virus
Wad_Medani_virus
Chobar_Gorge_virus
Big_Cypress_virus
Sathuvachari_virus
Skunk_River_virus
UMAV_2015_Australia_Mosquito
UMAV_1969_USA_Mosquito
KHV_2011_Japan_Mosquito
UMAV_2019_Germany_Blackbird
UMAV_2019_Germany_Greattit2
UMAV_2019_Germany_Bluetit1
Corriparta_virus
Parrys_Lagoon_virus
Kammavanpettai_virus
Bluetongue_virus
EHDV
Eubenangee_virus
Wallal_virus
Changuinola_virus
Warrego_virus
Lebombo_virus
Orungo_virus
EEV
African_horse_sickness_virus
Palyam_virus
St_Croix_River_virus
OCP1
0.6
KHV_2011_Japan_Mosquito
UMAV_2019_Germany_Bluetit1
UMAV_2019_Germany_Blackbird
UMAV_1969_USA_Mosquito
UMAV_2019_Germany_Greattit2
UMAV_2015_Australia_Mosquito
Kammavanpettai_virus
Corriparta_virus
Parrys_Lagoon_virus
Big_Cypress_virus
Sathuvachari_virus
Skunk_River_virus
CHeRI_orbivirus_3-1
Yunnan_orbivirus
Guangxi_orbivirus
CHeRI_orbivirus_1
CHeRI_orbivirus_2-1
Mobuck_virus
Peruvian_Horse_Sickness_orbivirus
Eubenangee_virus
Wallal_virus
Changuinola_virus
Warrego_virus
Bluetongue_virus
EHDV
Lebombo_virus
Orungo_virus
AHSV
Palyam_virus
EEV
Kemerovo_virus
Tribec_virus
Great_Island_virus
Wad_Medani_virus
Chenuda_virus
Chobar_Gorge_virus
St_Croix_River_virus
VP4
0.3
KHV_2011_Japan_Mosquito
UMAV_2015_Australia_Mosquito
UMAV_2019_Germany_Blackbird
UMAV_2019_Germany_Bluetit1
UMAV_2019_Germany_Greattit2
UMAV_1969_USA_Mosquito
Kammavanpettai_virus
Corriparta_virus
Parrys_Lagoon_virus
CHeRI_orbivirus_1
CHeRI_orbivirus_2-1
Guangxi_orbivirus
PHS_orbivirus
Mobuck_virus
Yunnan_orbivirus
CHeRI_orbivirus_3-1
Big_Cypress_virus
Skunk_River_virus
Sathuvachari_virus
Bluetongue_virus
EHDV
Eubenangee_virus
Wallal_virus
Changuinola_virus
Warrego_virus
Orungo_virus
Lebombo_virus
Palyam_virus
African_horse_sickness_virus
EEV
Kemerovo_virus
Tribec_virus
Great_Island_virus
Chenuda_virus
Wad_Medani_virus
Chobar_Gorge_virus
St_Croix_River_virus

Figure S2 (1/3)

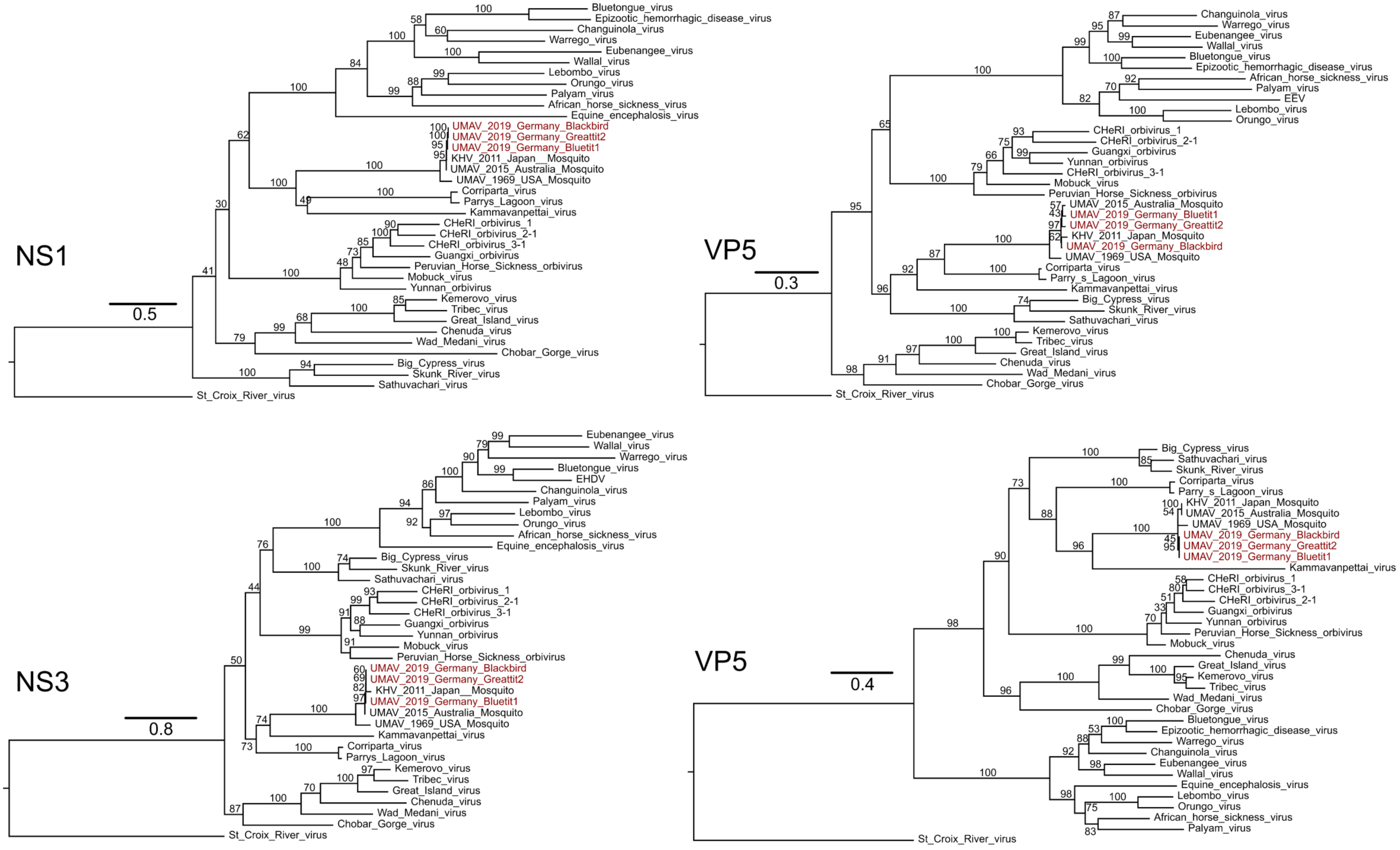
NS1
0.5
Bluetongue_virus
Epizootic_hemorrhagic_disease_virus
Changuinola_virus
Warrego_virus
Eubenangee_virus
Wallal_virus
Lebombo_virus
Orungo_virus
Palyam_virus
African_horse_sickness_virus
Equine_encephalosis_virus
UMAV_2019_Germany_Blackbird
UMAV_2019_Germany_Greattit2
UMAV_2019_Germany_Bluetit1
KHV_2011_Japan__Mosquito
UMAV_2015_Australia_Mosquito
UMAV_1969_USA_Mosquito
Corriparta_virus
Parrys_Lagoon_virus
Kammavanpettai_virus
CHeRI_orbivirus_1
CHeRI_orbivirus_2-1
CHeRI_orbivirus_3-1
Guangxi_orbivirus
Peruvian_Horse_Sickness_orbivirus
Mobuck_virus
Yunnan_orbivirus
Kemerovo_virus
Tribec_virus
Great_Island_virus
Chenuda_virus
Wad_Medani_virus
Chobar_Gorge_virus
Big_Cypress_virus
Skunk_River_virus
Sathuvachari_virus
St_Croix_River_virus
VP5
0.3
Changuinola_virus
Warrego_virus
Eubenangee_virus
Wallal_virus
Bluetongue_virus
Epizootic_hemorrhagic_disease_virus
African_horse_sickness_virus
Palyam_virus
EEV
Lebombo_virus
Orungo_virus
CHeRI_orbivirus_1
CHeRI_orbivirus_2-1
Guangxi_orbivirus
Yunnan_orbivirus
CHeRI_orbivirus_3-1
Mobuck_virus
Peruvian_Horse_Sickness_orbivirus
UMAV_2015_Australia_Mosquito
UMAV_2019_Germany_Bluetit1
UMAV_2019_Germany_Greattit2
KHV_2011_Japan_Mosquito
UMAV_2019_Germany_Blackbird
UMAV_1969_USA_Mosquito
Corriparta_virus
Parry_s_Lagoon_virus
Kammavanpettai_virus
Big_Cypress_virus
Skunk_River_virus
Sathuvachari_virus
Kemerovo_virus
Tribec_virus
Great_Island_virus
Chenuda_virus
Wad_Medani_virus
Chobar_Gorge_virus
St_Croix_River_virus
NS3
0.8
Eubenangee_virus
Wallal_virus
Warrego_virus
Bluetongue_virus
EHDV
Changuinola_virus
Palyam_virus
Lebombo_virus
Orungo_virus
African_horse_sickness_virus
Equine_encephalosis_virus
Big_Cypress_virus
Skunk_River_virus
Sathuvachari_virus
CHeRI_orbivirus_1
CHeRI_orbivirus_2-1
CHeRI_orbivirus_3-1
Guangxi_orbivirus
Yunnan_orbivirus
Mobuck_virus
Peruvian_Horse_Sickness_orbivirus
UMAV_2019_Germany_Blackbird
UMAV_2019_Germany_Greattit2
KHV_2011_Japan__Mosquito
UMAV_2019_Germany_Bluetit1
UMAV_2015_Australia_Mosquito
UMAV_1969_USA_Mosquito
Kammavanpettai_virus
Corriparta_virus
Parrys_Lagoon_virus
Kemerovo_virus
Tribec_virus
Great_Island_virus
Chenuda_virus
Wad_Medani_virus
Chobar_Gorge_virus
St_Croix_River_virus
VP5
0.4
Big_Cypress_virus
Sathuvachari_virus
Skunk_River_virus
Corriparta_virus
Parry_s_Lagoon_virus
KHV_2011_Japan_Mosquito
UMAV_2015_Australia_Mosquito
UMAV_1969_USA_Mosquito
UMAV_2019_Germany_Blackbird
UMAV_2019_Germany_Greattit2
UMAV_2019_Germany_Bluetit1
Kammavanpettai_virus
CHeRI_orbivirus_1
CHeRI_orbivirus_3-1
CHeRI_orbivirus_2-1
Guangxi_orbivirus
Yunnan_orbivirus
Peruvian_Horse_Sickness_orbivirus
Mobuck_virus
Chenuda_virus
Great_Island_virus
Kemerovo_virus
Tribec_virus
Wad_Medani_virus
Chobar_Gorge_virus
Bluetongue_virus
Epizootic_hemorrhagic_disease_virus
Warrego_virus
Changuinola_virus
Eubenangee_virus
Wallal_virus
Equine_encephalosis_virus
Lebombo_virus
Orungo_virus
African_horse_sickness_virus
Palyam_virus
St_Croix_River_virus

Figure S2 (2/3)

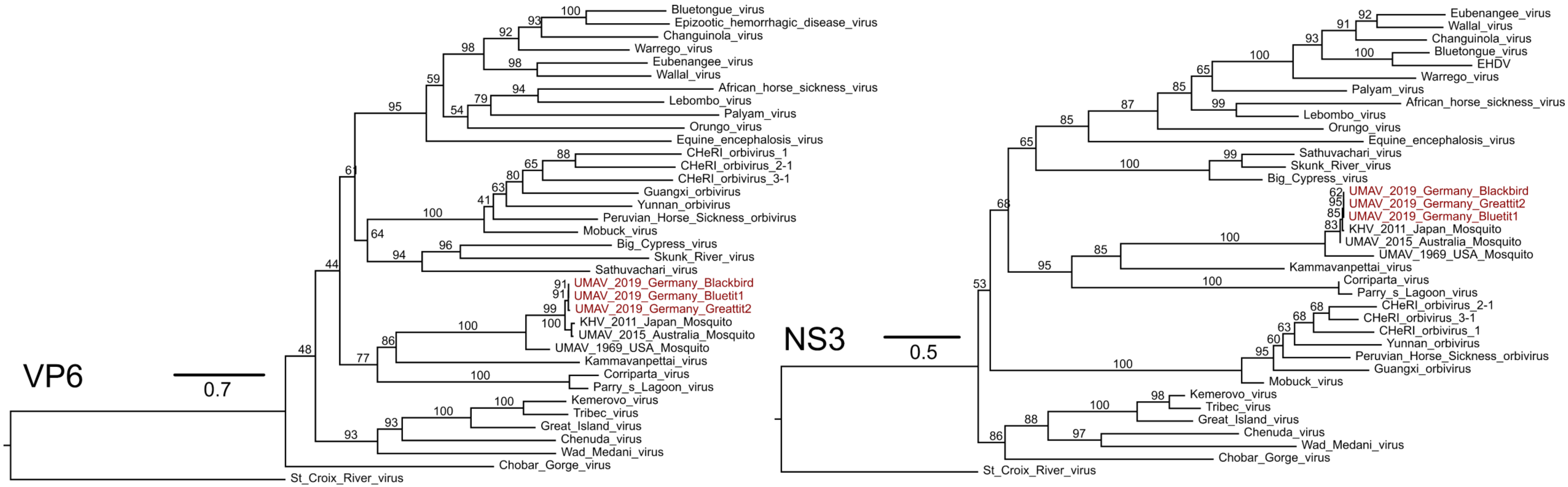

Figure S2 (3/3). Maximum likelihood phylogenetic trees of members of the genus *Orbivirus* using protein sequences from ten segments. Red text indicates genomes acquired in this study. Abbreviations: EEV – Equine encephalosis virus; EHDV – Epizootic hemorrhagic disease virus; AHSV – African Horse sickeness virus; PHS orbivirus – Peruvian Horse Sickness orbivirus

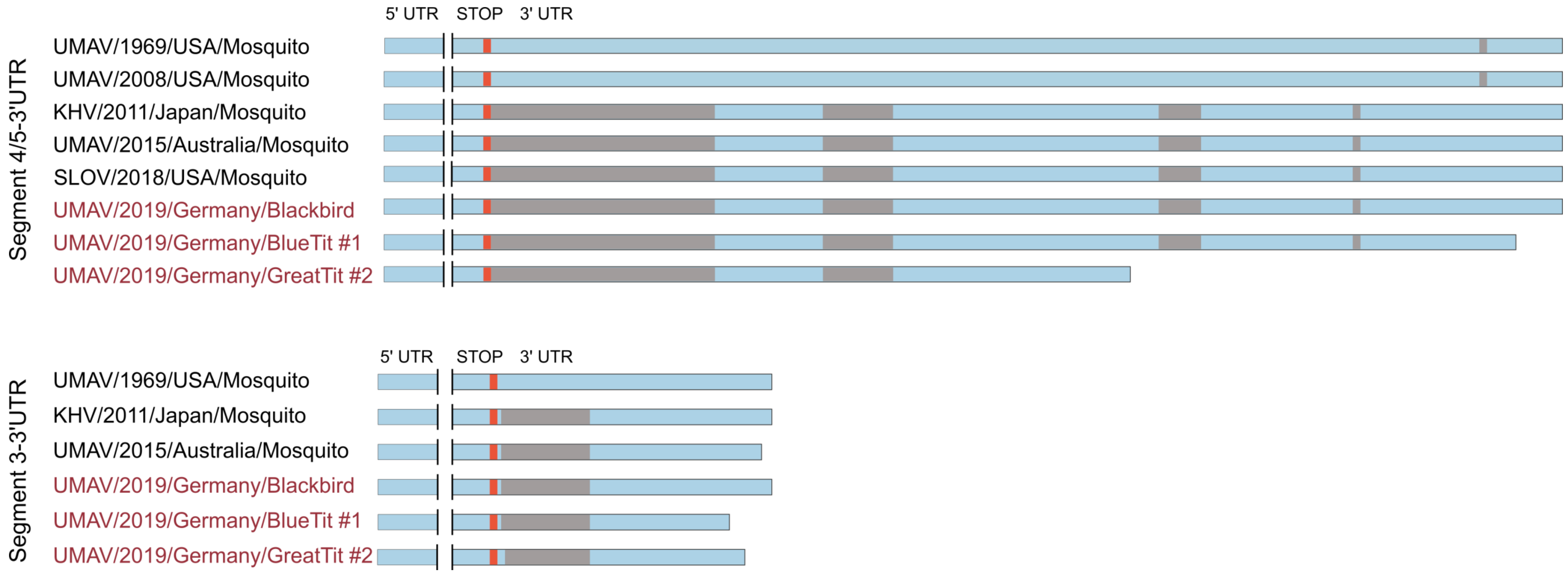

Figure S3. Schematic diagram of alignments of the 3’ untranscribed regions (UTR) of UMAV segments that encode the NS1 gene (Above) and OCP1 gene (Below). Red texts indicate UMAV variants detected in this study. Blue, red, and grey boxes indicate aligned sequence, stop codon position (STOP), and gaps. The nucleotide sequences of the UMAV/2008/USA /Mosquito and SLOV/2018/USA/Mosquito segment 3 were not available in the database. The number below the gray boxes indicated the number of deleted nucleotides.

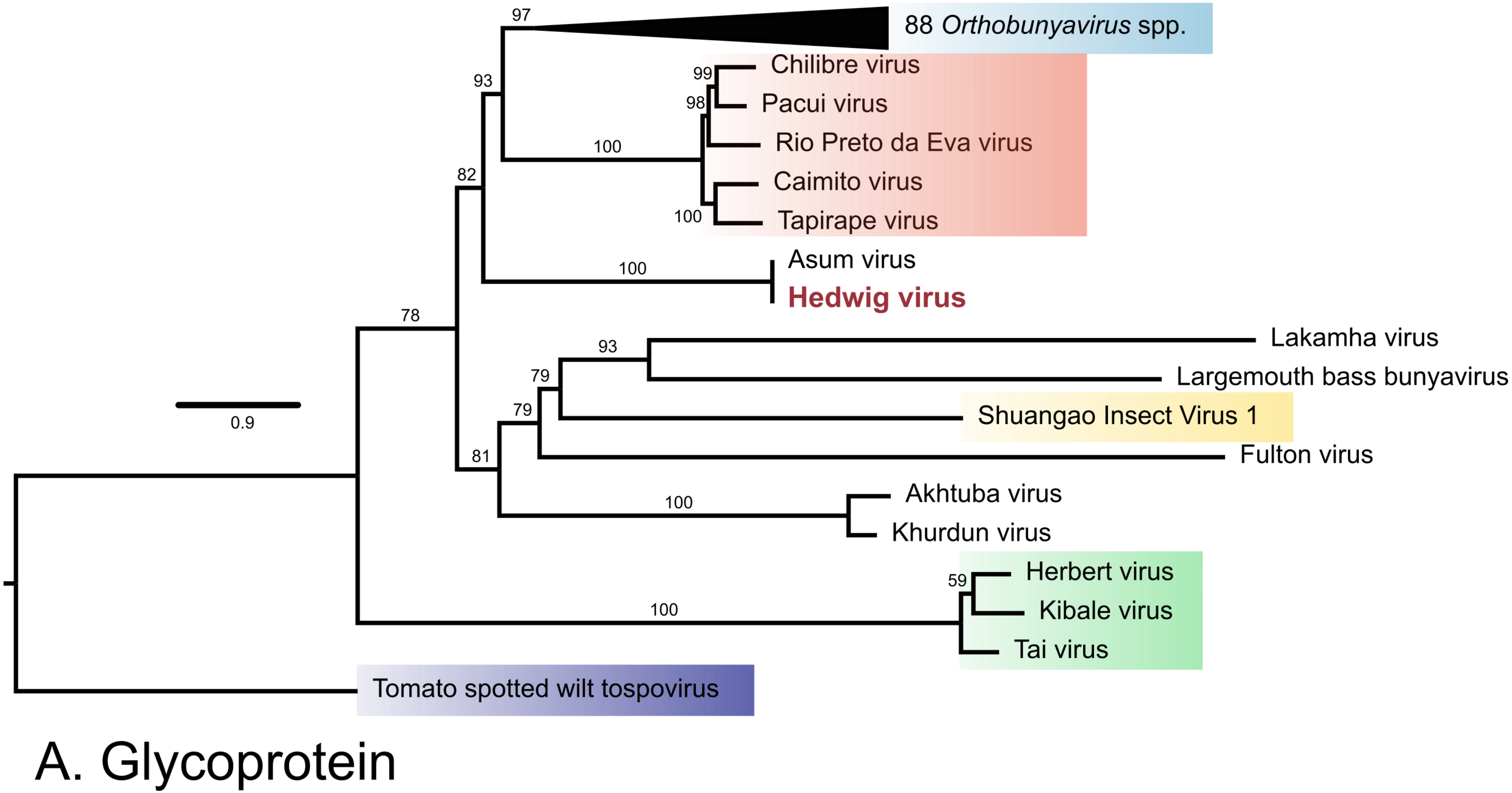
97
88 *Orthobunyavirus* spp.
93
99
Chilibre virus
98
Pacui virus
100
Rio Preto da Eva virus
82
Caimito virus
100
Tapirape virus
100
Asum virus
Hedwig virus
78
93
Lakamha virus
79
Largemouth bass bunyavirus
0.9
79
Shuangao Insect Virus 1
81
Fulton virus
100
Akhtuba virus
Khurdun virus
59
Herbert virus
100
Kibale virus
Tai virus
Tomato spotted wilt tospovirus
A. Glycoprotein

Figure S4 (1/2).

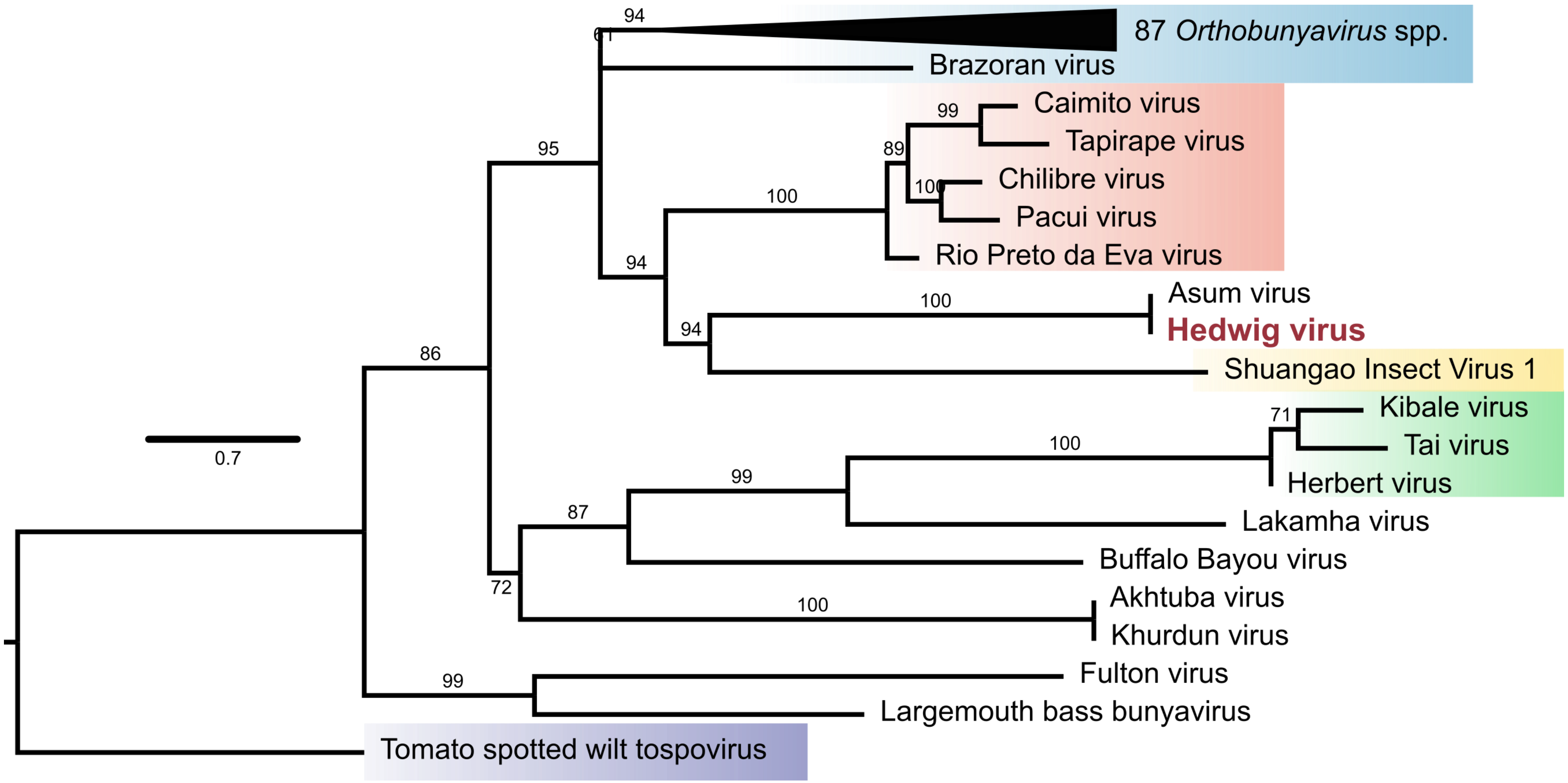

B. Nucleocapsid.

Figure S4 (2/2). Maximum likelihood trees of the the glycoprotein precursors (A) and nucleocapsid (B) of representative peribunyaviruses, Asum virus and Hedwig virus (printed red). Blue, orange, yellow, green, and uncolored represent the corresponding genera: *Orthobunyavirus, Pacuvirus, Shangavirus, Herbevirus*, and unclassified *Peribunyaviridae*. (A) Maximum likelihood tree of the RdRp amino acid sequences. Ultrafast bootstrap analyses with 100,000 replicates supported the tree topology. Representative *Orthobunyavirus species* were collapsed into a triangle. Tomato spotted wilt tospovirus was used as an outgroup (violet). Accession numbers of available amino acid sequences from representative members of the family *Peribunyaviridae* and the outlier strain are indicated in Table S6.
